# Guinier peak analysis for visual and automated inspection of small-angle X-ray scattering data

**DOI:** 10.1107/S1600576716010906

**Published:** 2016-08-04

**Authors:** Christopher D. Putnam

**Affiliations:** a Ludwig Institute for Cancer Research, Department of Medicine, University of California School of Medicine, San Diego, 9500 Gilman Drive, La Jolla, CA 92093-0669, USA

**Keywords:** small-angle X-ray scattering, sample characterization, Guinier analysis, Guinier peak analysis, elongation ratio

## Abstract

The Guinier region in small-angle X-ray scattering (SAXS) defines the radius of gyration, *R*_g_, and the forward scattering intensity, *I*(0). In Guinier peak analysis (GPA), the plot of *qI*(*q*) *versus q* ^2^ transforms the Guinier region into a characteristic peak for visual and automated inspection of data. Deviations of the peak position from the theoretical position in dimensionless GPA plots can suggest parameter errors, problematic low-resolution data, some kinds of intermolecular interactions or elongated scatters. To facilitate automated analysis by GPA, the elongation ratio (ER), which is the ratio of the areas in the pair-distribution function *P*(*r*) after and before the *P*(*r*) maximum, was characterized; symmetric samples have ER values around 1, and samples with ER values greater than 5 tend to be outliers in GPA analysis. Use of GPA+ER can be a helpful addition to SAXS data analysis pipelines.

## Abstract 2

Guinier peak analysis (GPA), derived from the Guinier approximation, transforms the Guinier region of small-angle X-ray scattering data into a characteristic peak that verifies the existence of the Guinier region in the data. Deviation of the Guinier peak position in dimensionless GPA plots can be a useful addition to sample characterization and parameter validation protocols.

## 1. Introduction   

Small-angle X-ray scattering (SAXS) data provide a number of parameters that give insights into the conformation of macromolecules in solution, including the radius of gyration *R*_g_, the volume of correlation, the Porod volume, the surface-to-volume ratio and the correlation length (Rambo & Tainer, 2013 ▸; Glatter & Kratky, 1982 ▸). *R*_g_ is a measure of the effective size of the sample and is primarily determined by one of two methods (Putnam *et al.*, 2007 ▸). In the first method, *R*_g_ is determined using the Guinier approximation (Guinier & Fourmet, 1955 ▸) for the low-resolution scattering (*qR*_g_ < 1.1, or *qR*_g_ < 1.3 for globular scatters):
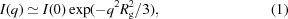
where *I*(*q*) is the scattering intensity, *I*(0) is the forward scattering intensity and the scattering vector magnitude *q* = (4π/λ)sinθ, θ being half the scattering angle and λ the wavelength of the incident radiation. *R*_g_ determined from the Guinier plot of ln[*I*(*q*)] *versus q* ^2^ is often termed the ‘reciprocal space’ *R*_g_. A lack of linearity in the Guinier plot is also an indicator of a lack of monodispersity and/or the presence of attractive or repulsive interactions between scatterers (Grant *et al.*, 2015 ▸; Jacques & Trewhella, 2010 ▸; Kikhney, 2010 ▸). In the second method, *R*_g_ is determined from the pair-distribution function *P*(*r*), which is a histogram of all inter-electron distances in the scattering particle:
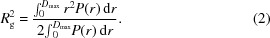

*D*_max_ is the maximum intraparticle distance. The *P*(*r*)-derived *R*_g_, also called the ‘real space’ *R_g_*, has the advantage of being derived from the entire scattering curve and not just the lowest-resolution data. The lowest-resolution data can be challenging to collect for samples with large *R*_g_ values or on beamlines with suboptimal positioning of the beam stop, parasitic scattering or beam divergence (Wignall *et al.*, 1990 ▸; Li *et al.*, 2012 ▸). Good agreement between the ‘reciprocal space’ and ‘real space’ *R*_g_ and *I*(0) values is often used as an indicator for a well measured dataset.

This article describes and demonstrates Guinier peak analysis (GPA), which provides a useful tool to validate the existence of the Guinier region, even when only a small quantity of data in the Guinier region has been collected. In conjunction with the elongation ratio (ER), which is a parameter that describes the asymmetry and non-compactness of the scattering based on the *P*(*r*) function, GPA can help to characterize SAXS samples and to validate refined parameters. A key advantage of the GPA+ER analysis is that only the raw scattering curve is required.

## 2. Methods   

### 2.1. Guinier peak analysis   

**Figure 1:**
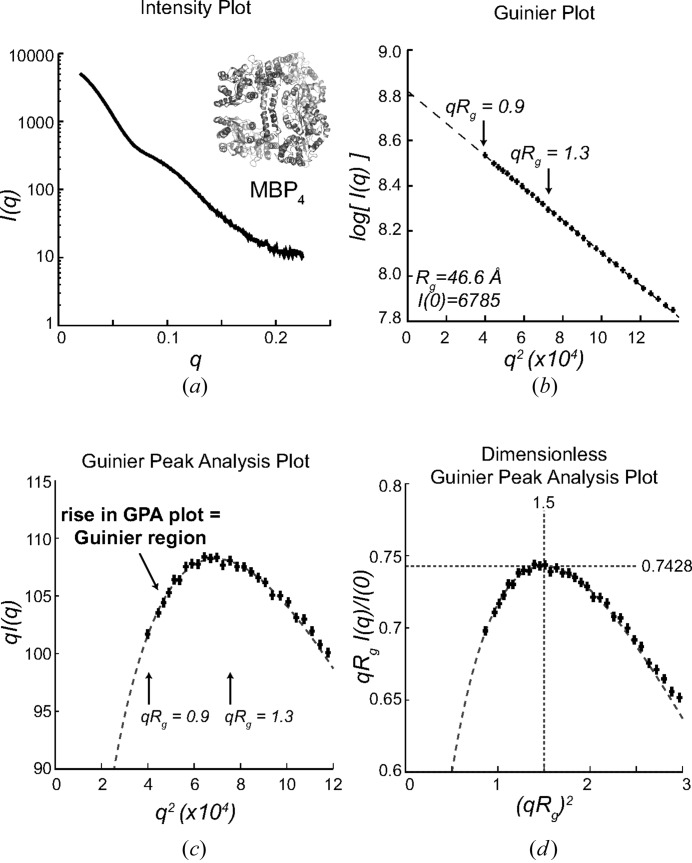
The GPA plot reveals the presence or absence of the Guinier region. (*a*) Scattering curve collected from a tetrameric maltose binding protein fusion protein (inset) (Mendillo *et al.*, 2007 ▸). (*b*) The traditional Guinier plot was linear, and the values *R*_g_ = 46.6 Å and *I*(0) = 6784.7 were extracted from the fit of the line (dashed) to the data (crosses) in the *qR*_g_ range of 0.9–1.3. (*c*) The GPA plot reveals that a small number of points in the Guinier region (crosses) were collected, as shown by the presence of the observed rise in the GPA plot, even without fitting the Guinier approximation (dashed line). Deviations of experimental data at higher resolutions from the Guinier approximation correspond to regions for which the Guinier approximation no longer applies. (*d*) The peak in the dimensionless GPA plot for this sample has a value (1.396, 0.7420), which is close to the theoretical position of (1.5, 0.7428).

A plot of *qI*(*q*) *versus q* ^2^ transforms the Guinier region into a peak (Fig. 1 ▸). This GPA plot can be derived by multiplying both sides of the Guinier approximation (1) by *q* to obtain
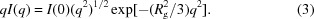
The GPA plot rises from *q* ^2^ values near zero to a theoretical maximum at *q*_max_^2^ = 1.5/*R*_g_^2^ or *q*_max_ *R*_g_ ≃ 1.22, and hence includes the Guinier region (*qR*_g_ < 1.0–1.3, depending on sample shape; see Supplementary Fig. 6). One variant of the GPA plot can be derived by taking the natural logarithm of (3) to yield
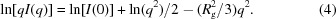
As logarithms are monotonically increasing functions, the peak in the ln[*qI*(*q*)] *versus q* ^2^ plot is also at 1.5/*R*_g_^2^. The plot derived from equation (4) is also used in the ‘modified Guinier analysis’ to determine the radius of gyration of the cross section of extended molecules at intermediate resolutions (Glatter & Kratky, 1982 ▸). Another variant of the GPA plot is *qI*(*q*) *versus q*, which has a theoretical maximum at *q*_max_ = (1.5^1/2^)/*R*_g_.

### 2.2. Dimensionless GPA   

The dimensionless version of the GPA plot is *qR*_g_ *I*(*q*)/*I*(0) *versus* (*qR*_g_)^2^. In the Guinier region, this plot follows the functional form
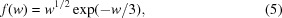
where *w* = (*qR*_g_)^2^. The Guinier approximation in the dimensionless GPA plot has a peak at (*qR*_g_)^2^ = 1.5 and *qR*_g_ *I*(*q*)/*I*(0) = (1.5)^1/2^exp(−0.5) = 0.7428.

This result indicates that the Guinier peak position in the normal GPA plot (*x*, *y*) can be used to validate values of *R*_g_ and *I*(0) derived from the Guinier plot or from integration of the *P*(*r*) function; note that using a smoothed *y* value for the GPA peak (see §2.4) improves the analysis. The dimensionless position (*x*′, *y*′) can be calculated by
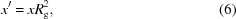


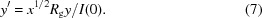
For the datasets in the BIOISIS (http://bioisis.net) and SASBDB (Valentini *et al.*, 2015 ▸) databases, the deviation of the (*x*′, *y*′) position from the theoretical position (1.5, 0.7428) was found to be sensitive to annotation errors in *R*_g_ and *I*(0) and/or to samples that defeat the heuristic for identifying Guinier peak position (Supplementary Table 1). To minimize the effect of outliers, statistical measures were performed using medians and median absolute deviations instead of means and standard deviations. Outliers were identified as samples whose Guinier peak position was 3 median absolute deviations or more (also called the Hampel identifier with *k* = 3) from the theoretical positions in either axis in the dimensionless GPA plot.

Similarly the Guinier peak position in the normal GPA plot (*x*, *y*) can also be used to estimate *R*_g_ and *I*(0):
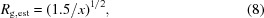


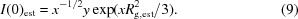
This estimate, however, is less precise than that derived from fitting the Guinier region in a traditional Guinier plot as it (i) is incorrect for elongated scatterers and (ii) is less accurate for more globular scatterers as it estimates the values only with data in the vicinity of *qR*_g_^2^ = 1.5.

### 2.3. Calculation of scattering from regular solids   

Theoretical scattering was calculated for simple geometric bodies using the form factors with the online *I*(*q*) function calculator (http://www.staff.tugraz.at/manfred.kriechbaum/xitami/java/iq.html).

### 2.4. Automated determination of the position of the Guinier peak   

In order to use GPA to validate *R*_g_ and *I*(0) values, the position of the Guinier peak in the *qI*(*q*) *versus q* ^2^ plot must be found independently of the transformed Guinier approximation. Thus, in the present work the GPA plot was analyzed using scale-space peak picking (Liutkus, 2015 ▸), which generates a ‘criterion’ score that identifies local maxima based on their ability to remain at or near maxima in the presence of successive rounds of smoothing. The position with the maximum criterion score often, but not always, corresponded to the global maximum of the GPA. To identify this peak, a heuristic was applied whereby each point in the curve was assigned two ranks corresponding to its position in the criterion scores, *r*_c_, and its position in *qI*(*q*) values, *r_qI_*, where a rank of 1 was the highest value. The position of the Guinier peak was taken to be the point with the minimum value of *r*_c_ × *r_qI_*. As the intensity at any point can be affected by noise, the *y* value of the peak was taken from a polynomial fit to the local region of the peak. A small number of analyzed datasets contained noise that defeated the peak identification heuristic and were initially flagged as outliers (Supplementary Table 1); these samples were re-processed after trimming noisy regions or after manual identification of the Guinier peak position.

### 2.5. The elongation ratio   

**Figure 2:**
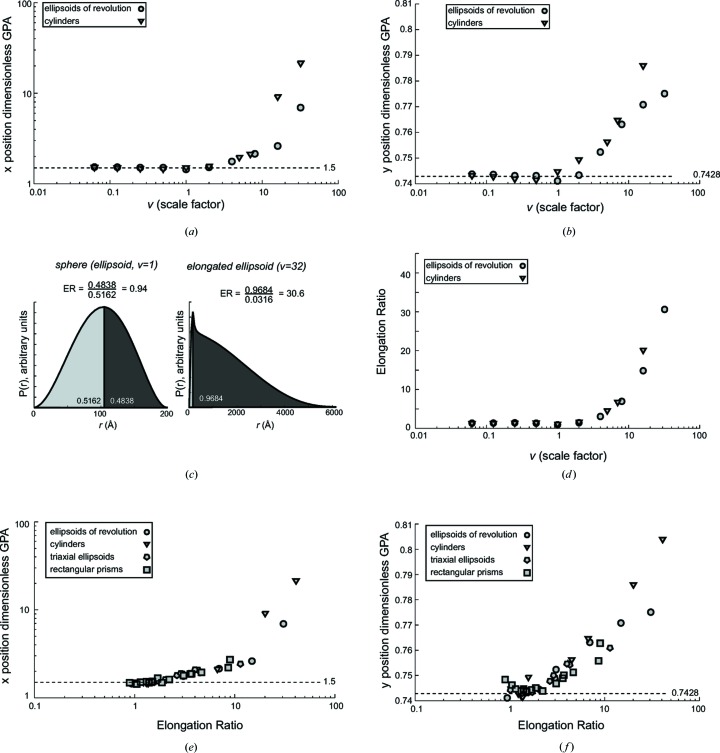
Characterization of the GPA plot and the ER. (*a*) The *x* positions of the dimensionless GPA peak in the (*qR*_g_)^2^ dimension for ellipsoids of revolution with axes *a*, *a*, *va* and cylinders with radius *r* and length *2vr* are plotted as a function of the scale factor *v*. (*b*) The *y* positions of the dimensionless GPA peak in the *qR*_g_ *I*(*q*)/*I*(0) dimension for ellipsoids of revolution and cylinders are plotted as a function of the scale factor *v*. (*c*) ER is the ratio of the areas under the *P*(*r*) function after and before the maximum. Relatively symmetric *P*(*r*) curves have ER values around 1.0, and *P*(*r*) curves from extended scatters have large ER values. (*d*) The ER values of ellipsoids and cylinders from panels (*a*) and (*b*) are plotted as a function of the scale factor *v*. (*e*) The *x* positions of the dimensionless GPA peak for ellipsoids of revolution, cylinders, triaxial ellipsoids and rectangular prisms are plotted as a function of ER. (*f*) The *y* positions of the dimensionless GPA peak for the regular solids from panel (*e*) are plotted as a function of ER.

A characteristic of *P*(*r*) functions from extended samples relative to *P*(*r*) functions from globular or hollow spheres is that the *P*(*r*) function reaches a maximum value at smaller values of *r*. ER is defined as the area under the *P*(*r*) function after the *P*(*r*) maximum divided by the area under the *P*(*r*) function prior to the *P*(*r*) maximum (Fig. 2 ▸ *c*):
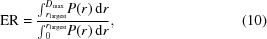
where *r*_largest_ is the value of *r* where the *P*(*r*) function reaches a maximum. This definition of the elongation ratio was found to be equivalent (differing only by a scaling constant) to other *P*(*r*)-based measures of elongation, such as the ratio of the weighted value of *r* after and before *r*_largest_ or the value of *R*_g_/*r*_largest_.

### 2.6. Derivation of *R_g_*-normalized *P*′(*r*′) functions from *P*(*r*) functions   

To compare scattering particle shape independent of size, *R*_g_-normalized forms of the *P*(*r*) functions, called here *P*′(*r*′) functions, were calculated. For each *R*_g_-normalized position *r_i_*′ = *r_i_*/*R*_g_, *P*′(*r_i_*′) was set equal to *P*(*r_i_*′*R*_g_). For the distance measurements described in §2.7, specific forms of the *P*′(*r*′) functions were generated in which the *P*(*r*) function was sampled in steps of *r*′ = 1/4 and scaled so that the sum of all sampled *P*′(*r*′) points was set to one. Importantly, the clustering analysis described in §2.7 was fairly insensitive to the precise sampling step size. Starting *P*(*r*) functions were taken from the BIOISIS and SASBDB databases, if available, or calculated from the deposited scattering using *GNOM* (Petoukhov *et al.*, 2012 ▸).

### 2.7. Clustering of size-sampled and normalized *P*′(*r*′) functions   

A distance between each pair of *P*′(*r*′) functions was calculated using a modified form of the composite angle distance (Putnam *et al.*, 2012 ▸). For each sampled point *r_i_*′ from *P_A_*′(*r*′) and *P_B_*′(*r*′), a two-dimensional vector **v** *_i_* was calculated. The *x* component of **v** *_i_* was the ‘shared component’ of *P_A_*′(*r_i_*′) and *P_B_*′(*r_i_*′), *i.e.* min[*P_A_*′(*r_i_*′), *P_B_*′(*r_i_*′)], and the *y* component of **v** *_i_* was the ‘unique component’, *i.e.* max[*P_A_*′(*r_i_*′), *P_B_*′(*r_i_*′)] − min[*P_A_*′(*r_i_*′), *P_B_*′(*r_i_*′)]. All of the vectors **v** *_i_* for each sampled point *r_i_*′ were then summed to generate the vector **v**_*A*,*B*_. The angle of **v**_*A*,*B*_ with the *x* axis, which could range from 0 to 90°, was calculated and scaled to be between 0 and 1. Identical *P*′(*r*′) functions had a distance of 0. *P*′(*r*′) functions lacking shared components at all sampled points, which is mathematically possible but physically unrealistic, had a distance of 1. All pairwise distances were then used to perform hierarchical agglomerative clustering using R (R Core Team, 2013 ▸).

## 3. Results   

### 3.1. Characteristics of the GPA plot   

The GPA plot of *qI*(*q*) *versus q* ^2^ provides two features that are useful to characterize SAXS datasets (Fig. 1 ▸). First, the rise in the GPA plot from *q* ^2^ = 0 to *q* ^2^ = *q*_max_^2^ provides evidence that the Guinier region is present in the dataset (Fig. 1 ▸ *c*). It can be challenging to confirm if data collection has successfully measured data from the Guinier region for samples that have large values of *R*_g_ and a small number of data points in that region. Importantly, the presence of the rise in the GPA plot does not require the fitting of any parameters and is readily identifiable by visual or automated inspection of the curve. Second, the position and value of the peak in the dimensionless GPA plot (Fig. 1 ▸ *d*), which is obtained by scaling with *R*_g_ and *I*(0), can be a useful tool to validate the *R*_g_ and *I*(0) values or help characterize scattering data (see §3.4).

To characterize the GPA plot, theoretical scattering was calculated from systematically varied ellipsoids of revolution and cylinders (Fig. 2 ▸). For all samples, the GPA rise validated the existence of the Guinier region in the calculated scattering (data not shown), and the *x* and *y* positions of the dimensionless GPA peak fell very close to the theoretical position of (1.5, 0.7428) except for elongated scatterers (Fig. 2 ▸ *a* and 2 ▸ *b*). These elongated scatterers were expected to be outliers in the dimensionless GPA analysis, as the Guinier approximation breaks down at *q* values before the Guinier peak at (*qR*_g_)^2^ = 1.5.

### 3.2. Characterization of samples by the elongation ratio   

The elongation ratio (§2.5; Fig. 2 ▸ *c*) was developed to facilitate quantitation of the elongation present in a scattering sample. The ER has two important advantages: (1) it can be applied to samples that cannot be described easily using simple geometric relationships, and (2) it is derived from the pair-distribution function and can be calculated independently of any real space model. For relatively symmetric objects, the ER value is around 1.0, whereas elongated cylinders and ellipsoids have ER values that are quite large (Fig. 2 ▸ *d*). For many different kinds of systematically varied regular solids, scatterers with large ER values are outliers in dimensionless GPA (Fig. 2 ▸ *e* and 2 ▸ *f*). Another measure of the utility of ER values for indicating asymmetry or flexibility is that the position of the peak in dimensionless Kratky plots (Durand *et al.*, 2010 ▸; Receveur-Brechot & Durand, 2012 ▸) is correlated with ER values (Supplementary Fig. 1).

### 3.3. Use of dimensionless GPA in identifying problematic scattering   

Samples with peaks in the dimensionless GPA plot that do not fall at the theoretical position describe a situation in which values derived from data in the vicinity of the peak disagree with the estimated values of *R*_g_ and *I*(0) from other techniques, often using lower-resolution data. These samples are expected to fall into one of four classes: (1) samples with problematic intensities in the Guinier region, (2) extended samples (see §3.2), (3) samples with errors in the estimated values of *R*_g_ and/or *I*(0) (see §3.4), and (4) samples with some forms of interparticle attractive (aggregation) or repulsive interactions.

To investigate the use of dimensionless GPA to identify samples in the last class, both simulated and experimental datasets were analyzed. Experimental scattering data (taken from the BIOISIS database) of glucose isomerase (GIKClP_1 and GNaClP_1) and lysozyme (LYKClP_1 and LNaClP_1) at low salt concentrations showed the characteristic features of interparticle repulsion (see *e.g.* Supplementary Fig. 2). These features included (i) a nonlinear Guinier region where the curves in the Guinier plot are concave downward, and (ii) local estimates of *R*_g_ and *I*(0) that increased with increasing values for the *q* ranges within the Guinier region. Moreover, these features were eliminated in scattering curves taken at higher salt concentrations, consistent with electrostatic repulsion. All of these samples had Guinier regions, as revealed by the rise in the GPA plots, but the dimensionless positions of the Guinier peaks identified these samples as problematic. Similarly, the calculated scattering from a polydisperse population of spheres had (i) a nonlinear Guinier region that was concave upward and (ii) local estimates of *R*_g_ and *I*(0) that decreased as the local *q* ranges increased in resolution (Supplementary Fig. 3). This calculated scattering had a Guinier region based on the rise in the GPA plots but was an outlier based on the dimensionless position of the Guinier peak. In these cases, dimensionless GPA analysis successfully identified these scattering curves as problematic.

In contrast, dimensionless GPA analysis was unable to identify other types of problematic samples. For example, theoretical scattering calculated from a mixture of *Thermus aquaticus* MutS monomers and dimers (PDB ID 1fw6; Junop *et al.*, 2001 ▸) at different ratios, which simulates a sample with heterogeneous assembly states, did not give rise to outliers in the dimensionless GPA plots (Supplementary Fig. 4); this is consistent with the fact that the observed *R*_g_^2^ in a heterogeneous solution is the *z* average of the *R*_g_^2^ values of the individual components. This Guinier region behavior makes it unsurprising that such samples are not outliers in GPA analysis. Consistently, GPA analysis of scattering from a bovine serum albumin sample taken before and after size-exclusion chromatography was unable to identify the problems in the pre-chromatographed sample despite a 9% increase in the observed *R*_g_ due to the presence of aggregates (Supplementary Fig. 5). These results indicate that substantial deviations in the dimensionless position for the Guinier peak are likely to be elongated or problematic and should be more carefully analyzed; however, agreement of the dimensionless GPA peak with theoretical values does not prove that scattering curves are suitable for structural analyses.

### 3.4. Application of the dimensionless GPA to experimental scattering   

**Figure 3:**
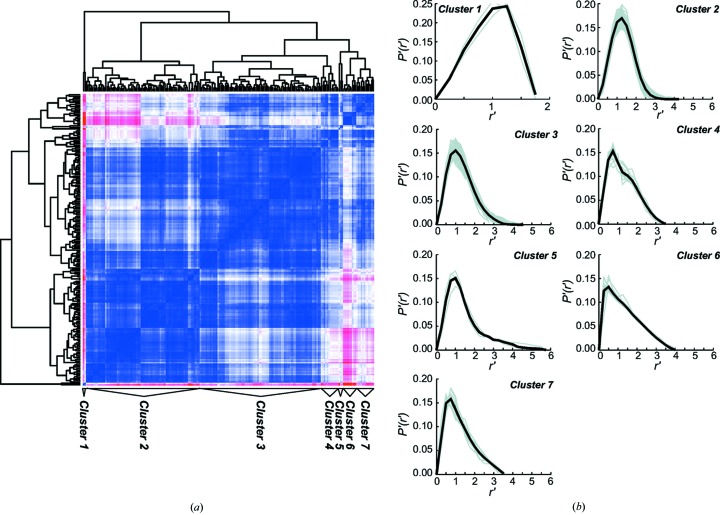
Clustering of 197 SAXS datasets by distances between *P*′(*r*′) functions. (*a*) Clustering of *R*_g_-sampled and -normalized *P*′(*r*′) function group scattering curves by the shape of the molecule. The distance matrix is colored from the most closely related pairs of *P*′(*r*′) functions in blue to the most distantly related pairs of *P*′(*r*′) functions in red. (*b*) Individual *P*′(*r*′) functions in each cluster are plotted in grey, and the average of all *P*′(*r*′) functions in each cluster is plotted in black.

To investigate the utility of dimensionless GPA in sample characterization, 197 scattering curves from the BIOISIS and SASBDB databases were analyzed (Supplementary Table 1). Since elongated samples are outliers (Fig. 2 ▸), the samples were first grouped by overall shape by hierarchical clustering (Fig. 3 ▸ *a*) using an *R*_g_-scaled version of the *P*(*r*) function that eliminated relative size differences [*P*′(*r*′)] functions; see §2.6). Cluster 1 contained hollow spheres (*e.g*. apo-ferritin); cluster 2 contained globular proteins with relatively symmetric *P*′(*r*′) functions (*e.g.* lysozyme); clusters 3–5 contained less symmetric globular proteins (*e.g.* the replication factor A DNA-binding core); cluster 6 contained very extended mol­lecules (*e.g.* repeats of surface protein G from *Staphylococcus aureus*); and cluster 7 contained somewhat extended mol­ecules like those in cluster 4 (*e.g.* the plakin domain of plectin) (Fig. 3 ▸ *b*).

**Figure 4:**
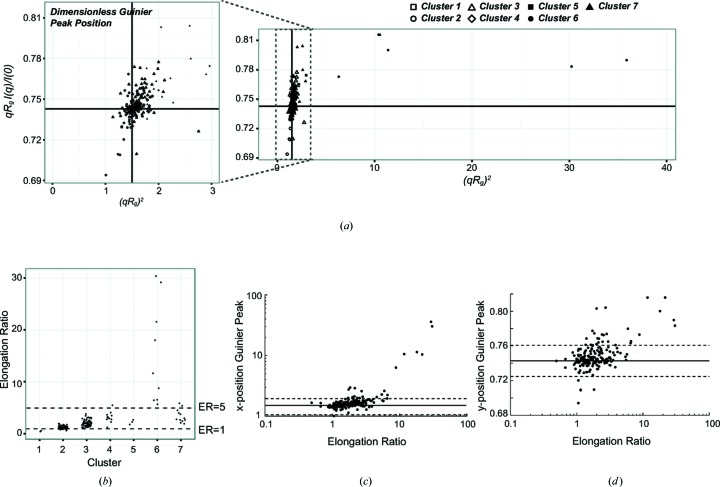
Position of the Guinier peak in the dimensionless GPA plots for all 197 experimental datasets. (*a*) The majority of samples fell in the vicinity of the theoretical position of (1.5, 0.428), and most outliers belonged to clusters 5, 6, and 7. (*b*) The distribution of ER values for samples in each cluster. Dashed lines are at ER values of 1.0 and 5.0. (*c*) The *x* positions of the Guinier peaks for the 197 datasets were correlated with the elongation ratio for the experimental datasets. The solid line corresponds to the theoretical position, and dashed lines are at ±3 MAD limits. (*d*) The *y* positions of the Guinier peaks were also correlated with the elongation ratio.

In the first round of analysis, most samples had peak positions in the dimensionless GPA plot that were near the theoretical values (Fig. 4 ▸ *a*). For the well behaved clusters 1–4, the (*qR*_g_)^2^ positions for the Guinier peaks had a median of 1.56 and a median absolute deviation (MAD) of 0.15. The *qR*_g_ *I*(*q*)/*I*(0) positions had a median of 0.744 and a MAD of 0.006. Outliers were identified as having deviations of the Guinier peak position in either dimension that were greater than 3 MAD values from the theoretical position. Annotation errors were found in 26 (13%) of the samples; these outliers were corrected by replacing the values after refitting Guinier plots (Supplementary Table 1). The identification of these errors suggests that GPA can provide a stringent check on the *R*_g_ and *I*(0) values. After correcting these annotation errors, the datasets were re-clustered and re-analyzed as described above.

As predicted from the breakdown of the Guinier approximation at *qR*_g_ < 1.22 for extended molecules, 89% of the datasets in cluster 6, which were measured from extended molecules, were outliers (Fig. 3 ▸ *c*). The median ER value for the symmetric *P*(*r*) functions in cluster 2 was 1.2, for the less symmetric *P*(*r*) functions in cluster 4 was 3.1, and for the elongated *P*(*r*) functions in cluster 6 was 15.4 (Fig. 4 ▸ *b*). These analyses suggest that outliers with ER > 5 are sufficiently elongated to be outliers in the GPA plot. There was also a clear correlation of ER with the Guinier peak position (Figs. 4 ▸ *c* and 4 ▸ *d*) as observed with the theoretical scatterers. To determine if ER values could predict the valid Guinier range, the 197 datasets were grouped on the basis of their ER values. The deviations of each scattering curve from the Guinier approximation within each group were then binned by (*qR*_g_)^2^, and the median and MAD were calculated for each bin. The maximum (*qR*_g_)^2^ bin with good agreement with the Guinier approximation was determined for each ER-based group. Datasets with ER < 4 had a maximum *qR*_g_ for the Guinier region well within the standard guideline of 1.3 for globular samples, whereas datasets with ER > 5 had a maximum *qR*_g_ for the Guinier region consistent with the standard guideline of 1.1 for extended samples (Supplementary Fig. 6).

## 4. Conclusions   

Measurement of data in the Guinier region is important for SAXS data collection. The GPA plot can confirm that these data have been collected, which is useful because data collection can be challenging for samples with large values of *R*_g_, and is well suited for both visual inspection and automated data analysis. In addition, the ER value provides a useful model-free method to quantitate how non-globular and compact a scatterer is to help guide analysis of dimensionless GPA results. Dimensionless GPA, when combined with the ER, is useful for rapidly evaluating the quality of SAXS datasets by identifying samples that are elongated, have incorrect *R*_g_ and/or *I*(0) values, exhibit problematic scattering in the Guinier peak region, and/or have some types of intermolecular attractive or repulsive interactions. Because the analyses are model-free and only require a scattering curve, the combination of GPA+ER is well suited for inclusion in SAXS analysis pipelines for identifying a subset of samples that require additional analysis.

## Supplementary Material

Supplementary figures and table in pdf format. DOI: 10.1107/S1600576716010906/vg5047sup1.pdf

Supplementary Table 1: excel spreadsheet for GPA analysis of 197 datasets. DOI: 10.1107/S1600576716010906/vg5047sup2.xlsx
