# Supplementary material for: Guinier peak analysis for visual and automated inspection of small-angle X-ray scattering data
Source: J Appl Crystallogr. 2016 Aug 4;49(Pt 5):1412–9. doi: 10.1107/S1600576716010906 (PMC5045725; doi:10.1107/S1600576716010906)
Supplement: Supplementary file 1 [file j-49-01412-sup1.pdf]

JOURNAL OF
APPLIED
CRYSTALLOGRAPHY

**Volume 49 (2016)**

**Supporting information for article:**

# Guinier Peak Analysis for Visual and Automated Inspection of Small Angle X-ray Scattering Data

**Christopher D. Putnam**

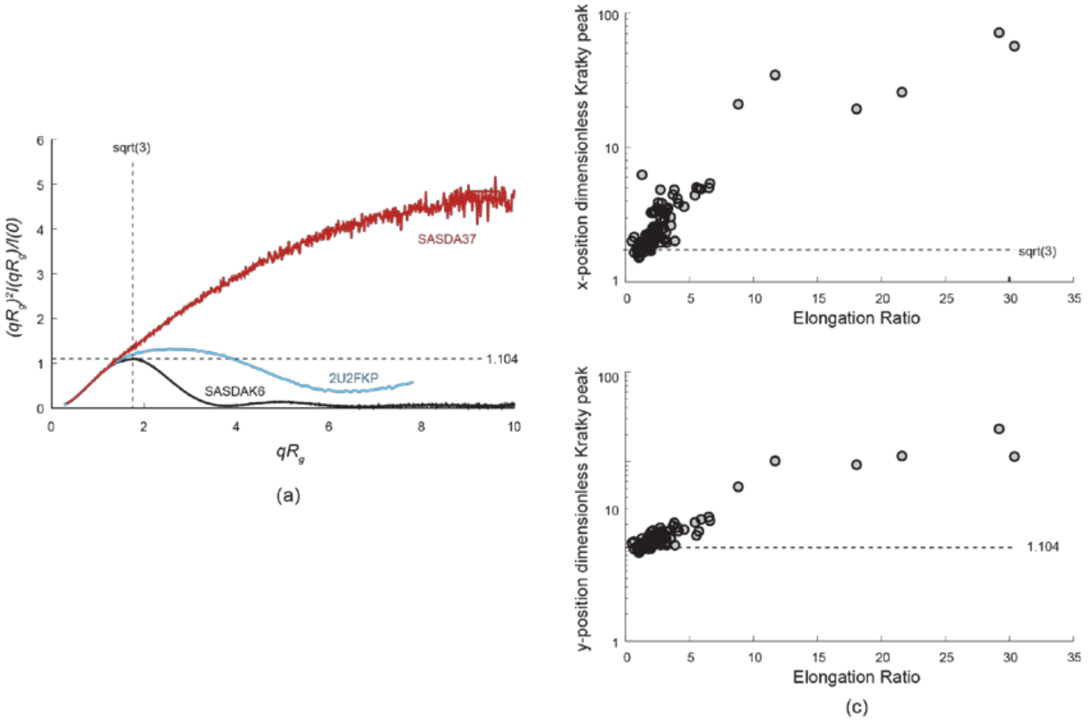


**Supplementary Figure 1. The ER value correlates with the peak position in dimensionless Kratky plots. (a)** The dimensionless Kratky plot (Durand *et al.*, 2010; Receveur-Brechot & Durand, 2012) has a peak at *( sqrt(3), 1.104 )* for compact, symmetric scatterers, such as SASDAK6 (*Streptomyces rubiginosus* xylose isomerase). Deviations from this position indicate either flexibility or asymmetry, such as with 2U2FKP (human U2AF65 RRM1-RRM3). Extended scatterers, such as SASDA37 (*Staphlococcus aureus* surface protein G, SasG construct containing repeats G51-G52), do not have a peak. Datasets are from the BIOSIS and SASBDB databases. (**b)** The x-positions of dimensionless Kratky peaks calculated for datasets in the BIOISIS and SASBDB databases were near the theoretical value (dashed line) for symmetric particles (ER near 1) and increased as the ER value increased. (**c)** Similar trends are observed for the y-positions of dimensionless Kratky peaks calculated for the datasets in BIOISIS and SASBDB.

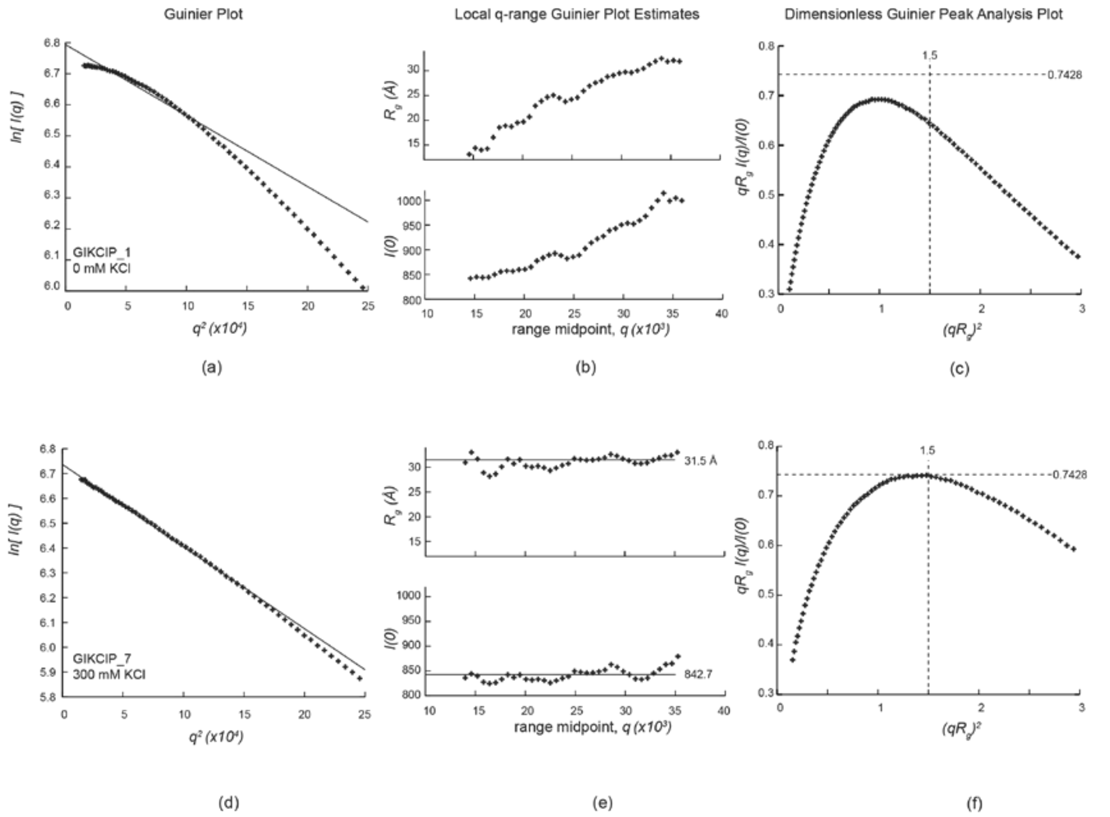


**Supplementary Figure 2. Glucose isomerase data with interparticle repulsion was an outlier in the dimensionless GPA plots.** Scattering data from *Streptomyces rubiginesis* glucose isomerase (BIOISIS dataset GIKCIP) contains data collected at 0 mM KCl (panels a-c) and at 300 mM KCl (panels d-f). **(a)** At low salt, the scattering showed evidence of interparticle repulsion due to the concave-down behaviour of the scattering in the Guinier plot. **(b)** Estimates of $R_g$ and *I(0)* based on local $q$-ranges containing six adjacent points within the Guinier region showed a continuous increase in $R_g$ and *I(0)* as the center of the $q$-range considered increased. **(c)** The Guinier peak in the dimensionless GPA plot was an outlier for the 0 mM KCl data. **(d)** At high salt, glucose isomerase had a linear Guinier region. **(e)** Local $q$-range estimates of $R_g$ and *I(0)* from the Guinier plot were consistent with each other and the global fit for the high salt data. **(f)** The dimensionless GPA plot revealed that the peak for the high salt data was very near to the theoretical position.

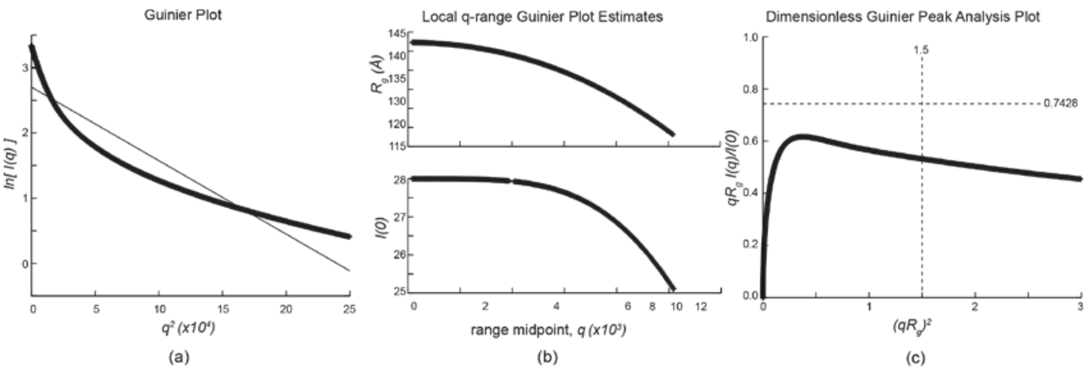


**Supplementary Figure 3. Scattering from a continuous distribution of spheres was an outlier in the dimensionless GPA plot. (a)** The Guinier plot of a calculated scattering continuous distribution of spheres of radii from 30 Å to 300 Å ($R_g$ 23.6 Å to 236 Å) revealed a concave upward distribution in the Guinier plot. **(b)** Estimates of $R_g$ and *I(0)* using 6 point *q*-ranges within the Guinier region decreased as the midpoint of the *q*-range increased. **(c)** The Guinier peak was a clear outlier in the dimensionless GPA plot.

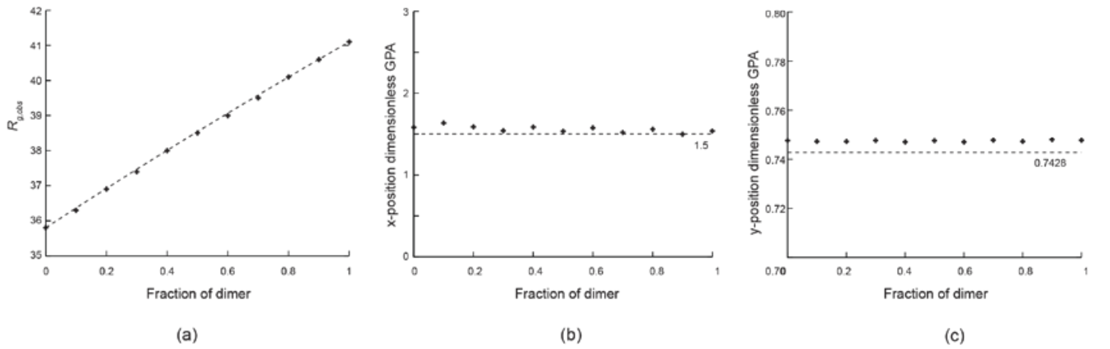


**Supplementary Figure 4. Scattering from theoretical mixtures of monomers and dimers were not outliers in dimensionless GPA plots. (a)** Theoretical mixtures of monomers and dimers calculated from the structure of *Thermus aquaticus* MutS (PDBid 1fw6, (Junop *et al.*, 2001)) using CRYSOL (Petoukhov *et al.*, 2012) give observed $R_g$ values (crosses) that z-averages of the monomer and dimer $R_g$ values. **(b)** The x-position of the Guinier peak in dimensionless GPA was insensitive to the dimer fraction. **(c)** The y-position of the Guinier peak in dimensionless GPA was insensitive to the dimer fraction.

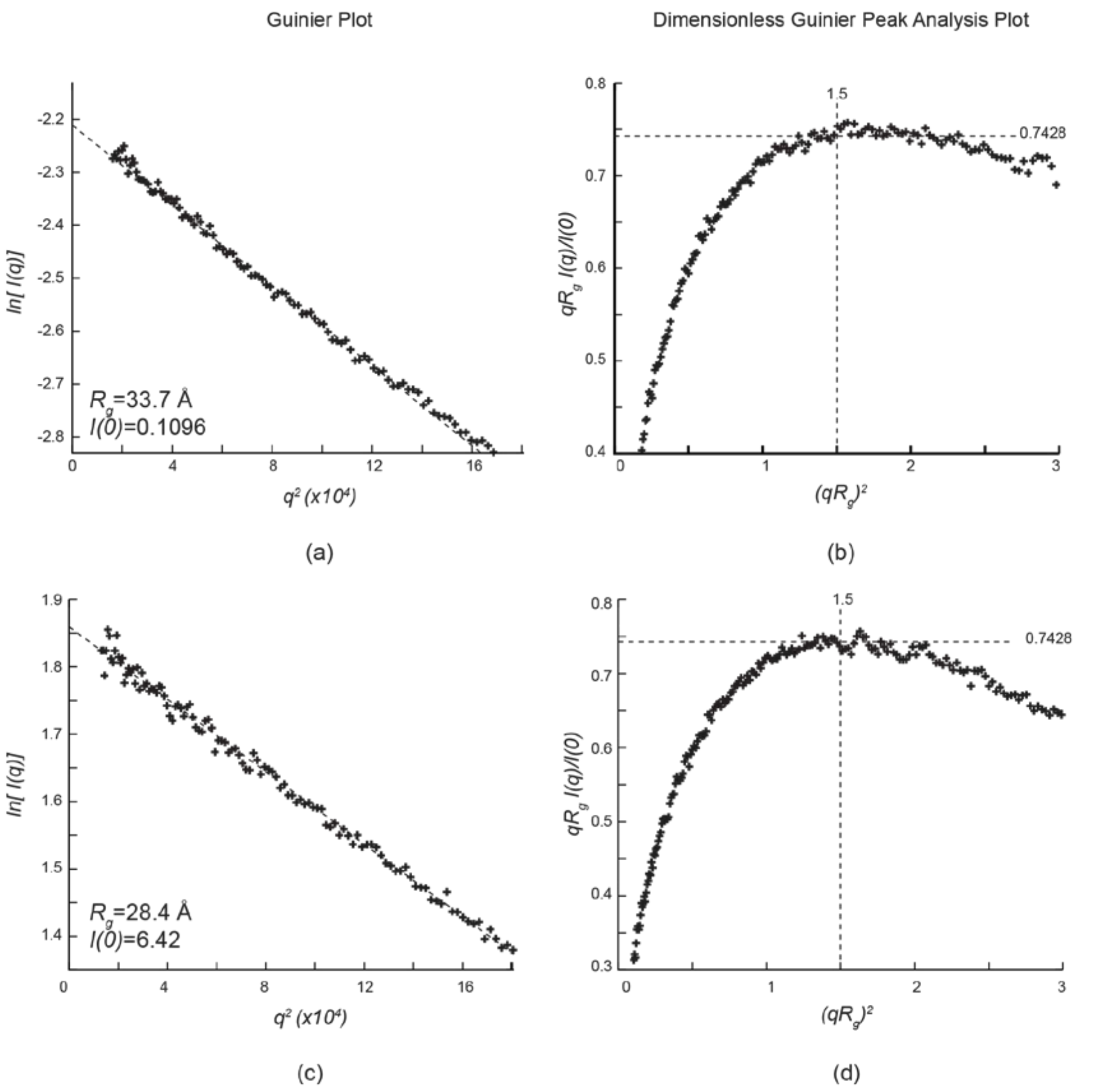


**Supplementary Figure 5. Scattering from bovine serum albumin (BSA) containing aggregation was not flagged as an outlier in GPA analysis.** BSA containing some aggregates (panels a and b) and BSA purified by size-exclusion chromatography (panels c and d) were analysed by SAXS. **(a)** The aggregated BSA had a linear Guinier region in the Guinier plot with an $R_g$=33.7 Å. **(b)** The Guinier peak in dimensionless GPA analysis was near the theoretical position. **(c)** BSA which had aggregates removed by size-exclusion chromatography also had a linear Guinier region with a lower $R_g$=28.4 Å. **(d)** The Guinier peak for the size-selected BSA was also near the theoretical position.

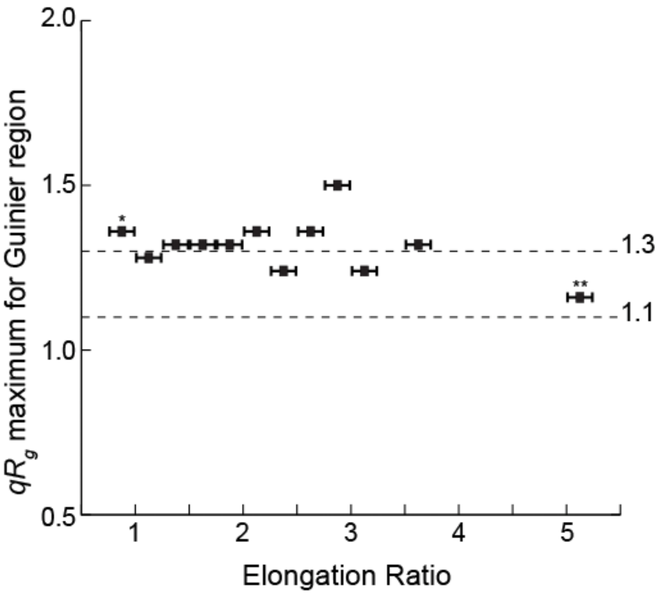


**Supplementary Figure 6. Maximum $qR_g$ range for the Guinier approximation as a function of ER.** After binning datasets based on the ER, the $qR_g$ maximum where the Guinier approximation held was determined (Supplementary Figures 2-4). The standard “rule-of-thumb” maxima of 1.3 (for globular scatterers) and 1.1 (for extended scatterers) are shown as dashed lines. The value shown in the 0.75-1.0 bind correspond to all datasets with ER values less than 1 (asterisk), and the value shown in the 5.0-5.25 bin correspond to all datasets with ER values greater than 5 (double asterisk).

**Supplementary Table 1. List of scattering curves from database sources and parameters (Excel spreadsheet).**

| *Cluster* | *Sample* | *Database* | *Rg, Angstroms (reported)* | *I(0) (reported)* | *Dmax, Angstroms (reported)* | *Fixed Rg/I(0)* | *Rg, Angstroms (used)* | *I(0) (used)* | *Peak (qRg)**2* | *Peak qRgI(q)/I(0)* | *Elongation Ratio* | *Guinier Peak Outlier* | *Notes* |
|---|---|---|---|---|---|---|---|---|---|---|---|---|---|
| 1 | SASDA82 | SASBDB | 67.5 | 356 | 128.0 | No | 67.5 | 356 | 1.6625 | 0.742652 | 0.4935 | No | |
| 1 | SASDAQ5 | SASBDB | 61.8 | 580240 | 155.0 | Yes (0.84, 0.81) | 86.4 | 884000 | 1.6440 | 0.744834 | 0.6739 | No | |
| 2 | MNME2P | BIOISIS | 37.2 | 39.8 | 138.0 | Yes (1.54, 0.000005) | 39.4 | 0.000264 | 1.7248 | 0.756392 | 1.1433 | No | |
| 2 | APSODP | BIOISIS | 20.5 | 20.5 | 65.0 | Yes (1.24, 15.7) | 20.7 | 438.1 | 1.2658 | 0.739752 | 1.3371 | No | |
| 2 | SASDAL2 | SASBDB | 25.0 | 37 | 78.0 | No | 25.0 | 37 | 1.774051 | 0.761184 | 1.3639 | **Yes** | |
| 2 | SASDB52 | SASBDB | 27.0 | 795 | 810.0 | No | 27.0 | 795 | 1.6987 | 0.753837 | 1.6106 | No | |
| 2 | SASDB72 | SASBDB | 24.0 | 623 | 750.0 | No | 24.0 | 623 | 1.4314 | 0.768532 | 1.3982 | **Yes** | Noisy high-resolution data required truncation before Guinier Peak Analysis |
| 2 | SASDAA8 | SASBDB | 18.7 | 87.52 | 50.0 | No | 18.7 | 87.52 | 1.3154 | 0.743641 | 1.1519 | No | |
| 2 | SASDA78 | SASBDB | 20.6 | 119.52 | 60.0 | No | 20.6 | 119.52 | 1.6685 | 0.746632 | 1.2167 | No | |
| 2 | SASDA92 | SASBDB | 41.0 | 222 | 109.0 | No | 41.0 | 222 | 1.6869 | 0.738675 | 1.2276 | No | |
| 2 | SASDAQ2 | SASBDB | 13.6 | 7.41 | 49.0 | No | 13.6 | 7.41 | 1.5596 | 0.738478 | 1.2102 | No | |
| 2 | LYSOZP_1 | BIOISIS | 15.6 | 57 | 49.0 | No | 15.6 | 57 | 1.5091 | 0.749424 | 1.1461 | No | |
| 2 | 1NADHP | BIOISIS | 23.1 | 701 | 96.0 | No | 23.1 | 701 | 1.5919 | 0.748508 | 1.3288 | No | |
| 2 | MRERAP | BIOISIS | 41.6 | 9.5 | 130.0 | No | 41.6 | 9.5 | 1.631885 | 0.749555 | 1.0517 | No | |
| 2 | HRCC1P_1 | BIOISIS | 25.1 | 61.2 | 80.0 | No | 25.1 | 61.2 | 1.624485 | 0.740717 | 1.3354 | No | |
| 2 | 1XYNTP_1 | BIOISIS | 16.7 | 133 | 43.0 | No | 16.7 | 133 | 1.4799 | 0.736812 | 1.1242 | No | |

| | | | | | | | | | | | | | | |
|---|---|---|---|---|---|---|---|---|---|---|---|---|---|---|
| 2 | SASDAX2 | SASBDB | 39.0 | 10.2 | 110.0 | No | 39.0 | 10.2 | 1.4133 | 0.742768 | 1.0252 | No | |
| 2 | SASDAK6 | SASBDB | 34.0 | 165.24 | 97.4 | No | 34.0 | 165.24 | 1.5255 | 0.741098 | 1.0918 | No | |
| 2 | 1TSPHP | BIOISIS | 16.7 | 4039 | 58.0 | No | 16.7 | 4039 | 1.6113 | 0.74614 | 1.2401 | No | |
| 2 | AT5GHP | BIOISIS | 21.2 | 0.03 | 74.0 | Yes (1.38, 0.78) | 21.7 | 0.0325 | 1.4458 | 0.736922 | 1.4249 | No | Evidence of intermolecular repulsion |
| 2 | SASDAX6 | SASBDB | 15.3 | 14.8 | 50.0 | No | 15.3 | 14.8 | 1.509618 | 0.743307 | 1.3696 | No | |
| 2 | 1PORHP | BIOISIS | 37.3 | 1102 | 133.0 | No | 37.3 | 1102 | 1.4696 | 0.744158 | 1.2948 | No | |
| 2 | SASDA96 | SASBDB | 15.0 | 779.5 | 47.9 | No | 15.0 | 779.5 | 1.4307 | 0.743226 | 1.1778 | No | |
| 2 | SASDA52 | SASBDB | 31.6 | 76.55 | 89.3 | No | 31.6 | 76.55 | 1.4812 | 0.742807 | 1.0545 | No | |
| 2 | SASDAU4 | SASBDB | 22.0 | 36.7 | 70.0 | Yes (1.38, 0.72) | 22.0 | 36.7 | 1.537475 | 0.741475 | 1.0642 | No | |
| 2 | 1RBDGP | BIOISIS | 11.0 | 1404 | 29.0 | Yes (1.46, 0.77) | 10.4 | 1410 | 1.304 | 0.729319 | 1.0726 | No | Sample defeated automated heuristic for identifying Guinier peak position |
| 2 | SASDA97 | SASBDB | 39.5 | 1380.54 | 105.0 | No | 39.5 | 1380.54 | 1.3831 | 0.738385 | 0.9115 | No | |
| 2 | SASDAK4 | SASBDB | 36.1 | 1.658 | 100.0 | No | 36.1 | 1.658 | 1.5056 | 0.743784 | 1.2265 | No | |
| 2 | 1PYR1P_1 | BIOISIS | 22.7 | 38.1 | 68.0 | No | 22.7 | 38.1 | 1.4616 | 0.739404 | 1.2397 | No | |
| 2 | SASDAA6 | SASBDB | 28.0 | 521.4 | 84.2 | No | 28.0 | 521.4 | 1.4738 | 0.742526 | 1.3094 | No | |
| 2 | SASDAH2 | SASBDB | 16.5 | 10.7 | 50.0 | No | 16.5 | 10.7 | 1.4169 | 0.743769 | 1.3646 | No | |
| 2 | SASDAK2 | SASBDB | 16.4 | 10.6 | 50.0 | No | 16.4 | 10.6 | 1.3998 | 0.746175 | 1.1607 | No | |
| 2 | SASDAC2 | SASBDB | 14.7 | 12.4 | 40.0 | No | 14.7 | 12.4 | 1.3949 | 0.745069 | 0.9952 | No | |
| 2 | SASDAG2 | SASBDB | 14.5 | 12.29 | 40.0 | No | 14.5 | 12.29 | 1.3572 | 0.741611 | 1.0575 | No | |
| 2 | BASODP | BIOISIS | 14.6 | 50 | 43.0 | No | 14.6 | 50 | 1.3631 | 0.756417 | 1.0705 | No | |
| 2 | 1GINTP_1 | BIOISIS | 33.4 | 372 | 96.0 | No | 33.4 | 372 | 1.4571 | 0.741787 | 1.1056 | No | |
| 2 | SASDAB2 | SASBDB | 13.0 | 7.46 | 35.0 | No | 13.0 | 7.46 | 1.433555 | 0.74042 | 0.9509 | No | |
| 2 | 1SPXGP | BIOISIS | 22.1 | 4540.2 | 80.0 | No | 22.1 | 4540.2 | 1.4470 | 0.733447 | 0.9587 | No | |
| 2 | SASDAB6 | SASBDB | 32.1 | 3733.8 | 91.0 | No | 32.1 | 3733.8 | 1.4682 | 0.741553 | 0.9947 | No | |
| 2 | 2SAMRR_1 | BIOISIS | 22.9 | 138 | 76.0 | No | 22.9 | 138 | 1.4835 | 0.748462 | 1.5118 | No | |
| 2 | SASDA35 | SASBDB | 25.0 | 45.524 | 85.0 | No | 25.0 | 45.524 | 1.625724 | 0.745803 | 1.7104 | No | |

| | | | | | | | | | | | | | |
|---|---|---|---|---|---|---|---|---|---|---|---|---|---|
| 2 | SASDAN3 | SASBDB | 47.0 | 10500 | 155.0 | Yes (1.84, 0.78) | 43.6 | 10300 | 1.5790 | 0.741906 | 1.4125 | No | Noisy high-resolution data required truncation before Guinier Peak Analysis |
| 2 | 1TLSRR | BIOISIS | 30.9 | 244 | 108.0 | No | 30.9 | 244 | 1.5149 | 0.743021 | 1.2330 | No | |
| 2 | SPNexP | BIOISIS | 36.1 | 212 | 123.0 | Yes (1.49, 0.76) | 35.2 | 216 | 1.4113 | 0.729042 | 1.2791 | No | |
| 2 | SASDAR6 | SASBDB | 56.7 | 188.32 | 179.0 | No | 56.7 | 188.32 | 1.6712 | 0.743945 | 1.4737 | No | |
| 2 | SS30SX | BIOISIS | 69.7 | 17800 | 231.0 | No | 69.7 | 17800 | 1.4594 | 0.741264 | 1.8138 | No | |
| 2 | SASDAS5 | SASBDB | 26.0 | 1032000 | 80.0 | No | 26.0 | 1032000 | 1.3068 | 0.729728 | 1.3970 | No | Sample defeated automated heuristic for identifying Guinier peak position |
| 2 | NTNH6P | BIOISIS | 39.5 | 1719.5 | 131.0 | No | 39.5 | 1719.5 | 1.4119 | 0.742183 | 1.5207 | No | |
| 2 | SASDAB7 | SASBDB | 33.7 | 0.05529 | 107.0 | No | 33.7 | 0.05529 | 1.4972 | 0.737327 | 1.2548 | No | |
| 2 | NTNH8P | BIOISIS | 39.7 | 1717.6 | 127.0 | No | 39.7 | 1717.6 | 1.5158 | 0.744404 | 1.6670 | No | |
| 2 | SASDA45 | SASBDB | 54.0 | 26.8 | 1650.0 | No | 54.0 | 26.8 | 1.5895 | 0.746588 | 1.6478 | No | |
| 2 | MNME3P | BIOISIS | 37.4 | 691 | 136.0 | No | 37.4 | 691 | 1.4773 | 0.729075 | 1.0902 | No | |
| 2 | MNME1P | BIOISIS | 38.6 | 39.8 | 139.0 | No | 38.6 | 39.8 | 1.5619 | 0.744014 | 1.0414 | No | Sample defeated automated heuristic for identifying Guinier peak position |
| 2 | SASDAV3 | SASBDB | 29.0 | 150.448 | 100.0 | No | 29.0 | 150.448 | 1.6353 | 0.747757 | 1.5035 | No | |
| 2 | ST2ILP | BIOISIS | 28.9 | 226 | 100.0 | No | 28.9 | 226 | 1.4865 | 0.742256 | 1.9956 | No | |
| 2 | SASDAC9 | SASBDB | 38.7 | 4298 | 164.0 | No | 38.7 | 4298 | 1.4691 | 0.742514 | 1.4479 | No | |
| 2 | SASDA32 | SASBDB | 29.2 | 59.3 | 93.0 | No | 29.2 | 59.3 | 1.4894 | 0.747321 | 1.2568 | No | |
| 2 | SASDA68 | SASBDB | 36.4 | 389.27 | 105.0 | No | 36.4 | 389.27 | 1.586519 | 0.745135 | 1.2416 | No | |
| 2 | SASDAM4 | SASBDB | 43.0 | 113 | 150.0 | No | 43.0 | 113 | 1.5124 | 0.748305 | 1.0723 | No | |
| 2 | SASDA62 | SASBDB | 42.1 | 164.38 | 127.0 | No | 42.1 | 164.38 | 1.3748 | 0.740794 | 1.0540 | No | |
| 2 | SASDAR5 | SASBDB | 26.0 | 123 | 80.0 | No | 26.0 | 123 | 1.4458 | 0.754706 | 1.3669 | No | |
| 2 | SASDAX4 | SASBDB | 28.0 | 62.3 | 94.0 | No | 28.0 | 62.3 | 1.448187 | 0.740658 | 1.3543 | No | |
| 2 | SASDA55 | SASBDB | 39.9 | 0.501 | 126.0 | No | 39.9 | 0.501 | 1.3056 | 0.729798 | 1.3970 | No | |
| 2 | SASDAL4 | SASBDB | 41.4 | 1.771 | 140.0 | No | 41.4 | 1.771 | 1.623549 | 0.742397 | 1.1181 | No | |

| | | | | | | | | | | | | | |
|---|---|---|---|---|---|---|---|---|---|---|---|---|---|
| 2 | SASDB62 | SASBDB | 25.0 | 717 | 900.0 | No | 25.0 | 717 | 1.3474 | 0.732413 | 1.5992 | No | Noisy high-resolution data required truncation before Guinier Peak Analysis |
| 2 | SASDAS3 | SASBDB | 19.2 | 41.5 | 60.0 | No | 19.2 | 41.5 | 1.512319 | 0.740467 | 1.4656 | No | |
| 2 | SASDAN2 | SASBDB | 15.8 | 13.5 | 50.0 | No | 15.8 | 13.5 | 1.4931 | 0.742121 | 1.2934 | No | |
| 2 | SASDAR2 | SASBDB | 16.0 | 13.65 | 50.0 | No | 16.0 | 13.65 | 1.5311 | 0.743298 | 1.4384 | No | |
| 2 | SASDA75 | SASBDB | 47.0 | 0.471 | 145.0 | No | 47.0 | 0.471 | 1.457558 | 0.731574 | 1.1232 | No | |
| 2 | 2HYPOP | BIOISIS | 35.6 | 92.58 | 110.0 | No | 35.6 | 92.58 | 1.4962 | 0.739943 | 1.5482 | No | |
| 2 | ZGDWKP | BIOISIS | 29.5 | 2.274 | 79.0 | No | 29.5 | 2.274 | 1.5540 | 0.746958 | 1.8074 | No | |
| 2 | GIKClP_1 | BIOISIS | 26.6 | 892.7 | 97.0 | No | 26.6 | 892.7 | 0.9895 | 0.69315 | 1.0567 | **Yes** | Intermolecular repulsion |
| 2 | GNaClP_1 | BIOISIS | 26.4 | 908.3 | 97.0 | No | 26.4 | 908.3 | 1.006675 | 0.693971 | 1.0567 | **Yes** | Intermolecular repulsion |
| 2 | LNaClP_1 | BIOISIS | 13.2 | 53.5 | 48.0 | No | 13.2 | 53.5 | 1.228378 | 0.707721 | 1.1461 | **Yes** | Intermolecular repulsion |
| 2 | LYKClP_1 | BIOISIS | 13.2 | 51.1 | 49.0 | No | 13.2 | 51.1 | 1.271802 | 0.708862 | 1.1461 | **Yes** | Intermolecular repulsion |
| 2 | SASDAD5 | SASBDB | 85.5 | 4070 | 280.0 | No | 85.5 | 4070 | 1.4180 | 0.752408 | 0.8695 | No | |
| 2 | SASDAJ6 | SASBDB | 52.0 | 144 | 170.0 | Yes (1.51, 0.72) | 52.3 | 140 | 1.5262 | 0.748841 | 0.9162 | No | |
| 2 | SASDAJ4 | SASBDB | 38.0 | 1.15 | 110.0 | No | 38.0 | 1.15 | 1.4501 | 0.73496 | 0.6721 | No | |
| 2 | SASDAF4 | SASBDB | 36.0 | 91.2 | 120.0 | No | 36.0 | 91.2 | 1.5089 | 0.744933 | 1.9862 | No | |
| 3 | 1ASRDP | BIOISIS | 19.2 | 61.94 | 80.0 | No | 19.2 | 61.94 | 1.694879 | 0.737418 | 1.4880 | No | |
| 3 | SASDA58 | SASBDB | 26.2 | 2964 | 106.0 | No | 26.2 | 2964 | 1.537948 | 0.746376 | 1.7406 | No | |
| 3 | 1FERHP | BIOISIS | 30.9 | 1233 | 101.0 | No | 30.9 | 1233 | 1.663098 | 0.743909 | 1.9735 | No | Noisy data in the Guinier region ~qRg = 1.5 |
| 3 | MnmG1P | BIOISIS | 42.3 | 0 | 139.0 | Yes (n/a, n/a) | 42.2 | 0.000193 | 1.4025 | 0.739381 | 1.7649 | No | |
| 3 | SASDA27 | SASBDB | 35.1 | 156.8 | 119.3 | No | 35.1 | 156.8 | 1.6464 | 0.757837 | 1.4804 | No | |
| 3 | SASDAV6 | SASBDB | 32.0 | 68 | 109.0 | No | 32.0 | 68 | 1.5754 | 0.760273 | 1.7272 | No | |
| 3 | SASDAG5 | SASBDB | 32.0 | 130 | 112.0 | Yes (1.63, 0.77) | 31.8 | 133 | 1.6102 | 0.743314 | 1.5913 | No | |
| 3 | SASDAZ6 | SASBDB | 31.1 | 88.44 | 108.7 | No | 31.1 | 88.44 | 1.5701 | 0.747027 | 1.5744 | No | |
| 3 | SASDAD8 | SASBDB | 26.4 | 0.188 | 111.0 | No | 26.4 | 0.188 | 1.6453 | 0.746845 | 1.8524 | No | |

| | | | | | | | | | | | | | |
|---|---|---|---|---|---|---|---|---|---|---|---|---|---|
| 3 | SASDAJ5 | SASBDB | 24.0 | 40.26 | 76.0 | No | 24.0 | 40.26 | 1.4521 | 0.740452 | 1.4325 | No | Noisy low-resolution data requiring truncation |
| 3 | SASDAV4 | SASBDB | 25.0 | 39.2 | 85.0 | No | 25.0 | 39.2 | 1.5098 | 0.73028 | 1.5523 | No | Noisy low-resolution data requiring truncation |
| 3 | SASDAW4 | SASBDB | 24.0 | 44.9 | 83.0 | No | 24.0 | 44.9 | 1.5961 | 0.75296 | 1.3260 | No | |
| 3 | SASDAA2 | SASBDB | 37.8 | 82.8 | 110.0 | No | 37.8 | 82.8 | 1.913996 | 0.739959 | 1.5669 | No | |
| 3 | SASDA98 | SASBDB | 78.6 | 2510.92 | 240.0 | No | 78.6 | 2510.92 | 1.6469 | 0.743533 | 1.6469 | No | |
| 3 | SASDAQ4 | SASBDB | 58.5 | 87600 | 230.0 | No | 58.5 | 87600 | 1.7095 | 0.744691 | 1.6145 | No | |
| 3 | SASDA72 | SASBDB | 32.4 | 93.43 | 144.0 | No | 32.4 | 93.43 | 1.6926 | 0.750993 | 1.6307 | No | |
| 3 | SASDAW6 | SASBDB | 18.7 | 17.2 | 64.0 | Yes (1.73, 0.77) | 18.3 | 17.2 | 1.6614 | 0.751258 | 1.9022 | No | |
| 3 | SASDAN5 | SASBDB | 21.4 | 5.2 | 85.0 | Yes (1.43, 0.70) | 23.6 | 5.26 | 1.7427 | 0.764818 | 2.4577 | **Yes** | |
| 3 | SASDAD4 | SASBDB | 51.0 | 280 | 190.0 | No | 51.0 | 280 | 1.567348 | 0.772554 | 1.7253 | **Yes** | Sample defeated automated heuristic for identifying Guinier peak position |
| 3 | SASDAR3 | SASBDB | 22.7 | 16.7 | 77.0 | No | 22.7 | 16.7 | 1.8747 | 0.7608 | 1.7470 | No | |
| 3 | SASDAZ5 | SASBDB | 39.0 | 3638 | 135.0 | No | 39.0 | 3638 | 1.529431 | 0.761691 | 2.1253 | **Yes** | Sample defeated automated heuristic for identifying Guinier peak position |
| 3 | MnmG2X | BIOISIS | 45.3 | 41 | 158.0 | No | 45.3 | 41 | 1.8292 | 0.755605 | 2.3114 | No | |
| 3 | SASDAA5 | SASBDB | 40.0 | 45.63 | 145.0 | No | 40.0 | 45.63 | 1.65288 | 0.773147 | 2.4053 | **Yes** | |
| 3 | SASDAV5 | SASBDB | 40.0 | 45.31 | 135.0 | No | 40.0 | 45.31 | 1.913541 | 0.768707 | 2.0589 | **Yes** | Noisy high-resolution data required truncation before Guinier Peak Analysis |
| 3 | RPADCP | BIOISIS | 38.8 | 327.39 | 171.0 | No | 38.8 | 327.39 | 1.8405 | 0.751463 | 2.2612 | No | |
| 3 | SASDAW3 | SASBDB | 38.0 | 211.49 | 140.0 | No | 38.0 | 211.49 | 1.7140 | 0.740194 | 1.9017 | No | |
| 3 | 1TPHER | BIOISIS | 24.3 | 71.2 | 98.0 | No | 24.3 | 71.2 | 1.7104 | 0.758461 | 2.2193 | No | |
| 3 | SASDA85 | SASBDB | 49.0 | 109.38 | 174.0 | No | 49.0 | 109.38 | 1.538108 | 0.733432 | 2.0958 | No | |
| 3 | SASDAY5 | SASBDB | 33.0 | 71.09 | 135.0 | No | 33.0 | 71.09 | 1.495454 | 0.740075 | 2.6408 | No | |
| 3 | 1ATPHP | BIOISIS | 80.0 | 983 | 300.0 | No | 80.0 | 983 | 1.5344 | 0.733233 | 2.0318 | No | |
| 3 | SASDAC5 | SASBDB | 56.5 | 80 | 190.0 | Yes (1.60, 0.77) | 54.3 | 80.3 | 1.4817 | 0.741018 | 2.1704 | No | Noisy high-resolution data required truncation before Guinier Peak Analysis |
| 3 | SASDAL5 | SASBDB | 33.0 | 45.6 | 106.0 | No | 33.0 | 45.6 | 1.562542 | 0.751594 | 2.4225 | No | |
| 3 | 2TIAKP | BIOISIS | 30.3 | 1.48 | 100.0 | No | 30.3 | 1.48 | 1.6884 | 0.764981 | 2.9826 | **Yes** | |

| | | | | | | | | | | | | | |
|---|---|---|---|---|---|---|---|---|---|---|---|---|---|
| 3 | SASDAF5 | SASBDB | 41.0 | 88 | 144.0 | No | 41.0 | 88 | 1.7961 | 0.741403 | 2.3297 | No | |
| 3 | 1SF1KP | BIOISIS | 30.2 | 120.3 | 105.0 | No | 30.2 | 120.3 | 1.5827 | 0.737833 | 2.0182 | No | |
| 3 | SASDAY4 | SASBDB | 30.0 | 1.62 | 104.0 | No | 30.0 | 1.62 | 1.6256 | 0.753509 | 3.1643 | No | |
| 3 | 1TIAKP | BIOISIS | 27.2 | 172 | 100.0 | No | 27.2 | 172 | 1.2838 | 0.737583 | 3.2107 | No | |
| 3 | SASDAC4 | SASBDB | 35.0 | 122 | 130.0 | Yes (1.83, 0.77) | 32.5 | 117 | 1.5778 | 0.744143 | 2.4545 | No | |
| 3 | SASDA25 | SASBDB | 20.0 | 0.194 | 71.0 | Yes (1.48, 0.77) | 20.4 | 0.197 | 1.5435 | 0.77381 | 2.8271 | **Yes** | |
| 3 | 2TM1KP | BIOISIS | 29.6 | 3.52 | 100.0 | No | 29.6 | 3.52 | 1.6468 | 0.746663 | 3.1378 | No | |
| 3 | 1U2FKP | BIOISIS | 31.4 | 1001 | 115.0 | Yes (1.42, 0.14) | 31.3 | 192.6 | 1.4153 | 0.743583 | 3.5279 | No | |
| 3 | SASDAR4 | SASBDB | 39.7 | 206 | 165.0 | No | 39.7 | 206 | 1.6185 | 0.746448 | 3.1685 | No | |
| 3 | 6HYPHP | BIOISIS | 58.2 | 1102.5 | 280.0 | No | 58.2 | 1102.5 | 1.7616 | 0.758572 | 2.9934 | No | |
| 3 | 2U2FKP | BIOISIS | 24.6 | 196.2 | 85.0 | No | 24.6 | 196.2 | 1.6360 | 0.745241 | 2.4572 | No | |
| 3 | SASDAU3 | SASBDB | 27.6 | 34.3 | 90.0 | No | 27.6 | 34.3 | 1.987162 | 0.772487 | 2.5508 | **Yes** | Possible aggregation |
| 3 | SASDA46 | SASBDB | 30.0 | 1800 | 100.0 | No | 30.0 | 1800 | 2.0888 | 0.755409 | 2.8290 | **Yes** | |
| 3 | SASDAC7 | SASBDB | 35.5 | 0.02362 | 125.0 | No | 35.5 | 0.02362 | 1.7941 | 0.752962 | 2.1771 | No | |
| 3 | SASDAA7 | SASBDB | 39.8 | 0.07466 | 160.0 | Yes (1.70, 0.76) | 39.6 | 0.0744 | 1.6786 | 0.761283 | 1.6314 | **Yes** | |
| 3 | SASDA95 | SASBDB | 40.0 | 71.4 | 145.0 | No | 40.0 | 71.4 | 1.5696 | 0.746533 | 1.6264 | No | |
| 3 | SASDAY6 | SASBDB | 35.8 | 112.4 | 121.4 | No | 35.8 | 112.4 | 1.7378 | 0.762116 | 1.9850 | **Yes** | |
| 3 | SASDAH5 | SASBDB | 43.0 | 58 | 145.0 | No | 43.0 | 58 | 1.7202 | 0.75699 | 1.6866 | No | |
| 3 | SASDAT4 | SASBDB | 28.0 | 49.3 | 97.0 | No | 28.0 | 49.3 | 1.5965 | 0.756121 | 1.6818 | No | |
| 3 | SASDAW5 | SASBDB | 35.0 | 78.3 | 115.0 | No | 35.0 | 78.3 | 1.7316 | 0.754853 | 2.0481 | No | Noisy high-resolution data required truncation before Guinier Peak Analysis |
| 3 | SASDAG7 | SASBDB | 26.9 | 8235 | 94.0 | No | 26.9 | 8235 | 1.5557 | 0.744839 | 3.1415 | No | |
| 3 | SASDAS4 | SASBDB | 28.5 | 43.3 | 107.0 | Yes (1.32, 0.70) | 30.4 | 43.17 | 1.587472 | 0.744794 | 2.0373 | No | |

| | | | | | | | | | | | | | | |
|---|---|---|---|---|---|---|---|---|---|---|---|---|---|---|
| 3 | SASDA84 | SASBDB | 22.5 | 170 | 80.0 | Yes (1.67, 0.81) | 20.5 | 171 | 1.6656 | 0.812158 | 1.7491 | **Yes** | Sample defeated automated heuristic for identifying Guinier peak position |
| 3 | SASDAT5 | SASBDB | 31.0 | 1158700 | 120.0 | Yes (1.86, 0.77) | 29.2 | 1130000 | 1.6482 | 0.74266 | 2.5741 | No | |
| 3 | 2HYPHP | BIOISIS | 17.1 | 35.27 | 60.0 | Yes (1.96, 0.78) | 15.4 | 33.3 | 1.5866 | 0.748622 | 1.4980 | No | |
| 3 | 5HYPHP | BIOISIS | 28.4 | 0.74 | 99.0 | Yes (2.19, 131) | 24.6 | 113.05 | 1.6440 | 0.747264 | 1.7268 | No | |
| 3 | 1LYSRR | BIOISIS | 30.5 | 183 | 108.0 | No | 30.5 | 183 | 1.661734 | 0.746091 | 1.6735 | No | |
| 3 | 1P4P6R_1 | BIOISIS | 29.6 | 379 | 110.0 | No | 29.6 | 379 | 1.5651 | 0.742906 | 1.5252 | No | |
| 3 | SASDAH4 | SASBDB | 29.0 | 0.378 | 90.0 | No | 29.0 | 0.378 | 1.2824 | 0.754232 | 2.3587 | No | |
| 3 | 2PSUKX | BIOISIS | 37.7 | 442.14 | 105.0 | No | 37.7 | 442.14 | 1.7411 | 0.75145 | 1.3032 | No | |
| 3 | SASDA94 | SASBDB | 26.5 | 98.1 | 85.0 | No | 26.5 | 98.1 | 1.5187 | 0.740274 | 1.3136 | No | |
| 3 | SASDAP5 | SASBDB | 29.0 | 11.3 | 95.0 | No | 29.0 | 11.3 | 1.4412 | 0.760932 | 3.8579 | **Yes** | |
| 3 | SASDAG4 | SASBDB | 46.0 | 121 | 150.0 | No | 46.0 | 121 | 1.599853 | 0.75468 | 1.0529 | No | |
| 3 | 1TIAKX | BIOISIS | 24.7 | 331 | 80.0 | No | 24.7 | 331 | 1.4621 | 0.742017 | 1.9811 | No | |
| 3 | SASDAT6 | SASBDB | 22.0 | 590 | 70.0 | No | 22.0 | 590 | 1.6346 | 0.743326 | 1.7352 | No | |
| 3 | SASDAE5 | SASBDB | 37.0 | 86.9 | 128.0 | No | 37.0 | 86.9 | 1.5121 | 0.753815 | 1.8257 | No | |
| 3 | BoNT6P | BIOISIS | 47.3 | 2513.6 | 153.0 | Yes (1.86, 0.81) | 43.6 | 2510 | 1.5820 | 0.74483 | 1.6448 | No | |
| 3 | BoNT8P | BIOISIS | 43.3 | 1009.3 | 150.0 | No | 43.3 | 1009.3 | 1.4613 | 0.743991 | 1.6984 | No | |
| 3 | C3bE2P | BIOISIS | 48.2 | 155 | 180.0 | No | 48.2 | 155 | 1.553172 | 0.739958 | 1.3834 | No | |
| 3 | C3bEfP | BIOISIS | 48.6 | 160 | 160.0 | Yes (1.58, 0.72) | 48.6 | 155.2 | 1.576162 | 0.744359 | 1.2462 | No | |
| 3 | DBC30Y | BIOISIS | 34.9 | 1028.1 | 116.0 | No | 34.9 | 1028.1 | 1.5947 | 0.744852 | 1.9962 | No | |
| 3 | C3BSAP | BIOISIS | 46.7 | 133 | 176.0 | No | 46.7 | 133 | 1.5997 | 0.742634 | 1.2780 | No | |
| 3 | C3BXXP | BIOISIS | 46.8 | 133 | 161.0 | No | 46.8 | 133 | 1.6004 | 0.742792 | 1.2395 | No | |
| 3 | SASDAB5 | SASBDB | 30.0 | 38.14 | 105.0 | No | 30.0 | 38.14 | 1.5859 | 0.709536 | 1.8236 | **Yes** | |

| | | | | | | | | | | | | | |
|---|---|---|---|---|---|---|---|---|---|---|---|---|---|
| 3 | 3LYSRR | BIOISIS | 29.7 | 183 | 108.0 | Yes (1.19, 0.54) | 29.0 | 130.9 | 1.1384 | 0.736788 | 1.7344 | No | |
| 3 | RBFSHP | BIOISIS | 24.0 | 3226 | 95.0 | Yes (1.63, 0.79) | 22.9 | 3260 | 1.4876 | 0.744379 | 1.7546 | No | |
| 3 | SASDAH6 | SASBDB | 31.0 | 47.4 | 100.0 | No | 31.0 | 47.4 | 1.5850 | 0.736363 | 2.1704 | No | |
| 3 | SASDAN4 | SASBDB | 21.0 | 107.587 | 70.0 | No | 21.0 | 107.587 | 1.5207 | 0.749344 | 2.1737 | No | |
| 3 | SASDAT3 | SASBDB | 21.0 | 98.309 | 65.0 | No | 21.0 | 98.309 | 1.6058 | 0.75813 | 2.1239 | No | |
| 4 | SASDAS7 | SASBDB | 85.0 | 249 | 300.0 | No | 85.0 | 249 | 1.7884 | 0.749406 | 3.7165 | No | |
| 4 | SASDAF6 | SASBDB | 42.9 | 0.172 | 147.0 | No | 42.9 | 0.172 | 1.8192 | 0.743617 | 5.5491 | No | |
| 4 | SASDAZ4 | SASBDB | 30.0 | 0.38 | 105.0 | Yes (1.66, 0.724) | 30.6 | 0.376 | 1.72 | 0.745718 | 1.2908 | No | |
| 4 | SASDAB8 | SASBDB | 60.2 | 1.07 | 200.0 | No | 60.2 | 1.07 | 1.6751 | 0.753025 | 4.1392 | No | |
| 4 | SASDAM5 | SASBDB | 42.0 | 3664 | 147.0 | No | 42.0 | 3664 | 1.8825 | 0.752219 | 3.5554 | No | |
| 4 | SASDAX5 | SASBDB | 59.0 | 435.06 | 190.0 | No | 59.0 | 435.06 | 1.6055 | 0.746565 | 2.3318 | No | |
| 4 | SASDAG6 | SASBDB | 29.2 | 0.093 | 95.0 | No | 29.2 | 0.093 | 1.5235 | 0.73786 | 3.1415 | No | |
| 4 | SASDA24 | SASBDB | 78.0 | 274 | 270.0 | No | 78.0 | 274 | 1.4546 | 0.744826 | 2.8313 | No | |
| 4 | SASDAQ3 | SASBDB | 78.0 | 274 | 280.0 | No | 78.0 | 274 | 1.4546 | 0.742858 | 2.9930 | No | |
| 4 | SASDAX3 | SASBDB | 85.0 | 301 | 290.0 | No | 85.0 | 301 | 1.8518 | 0.742074 | 3.3326 | No | |
| 4 | SASDAY3 | SASBDB | 83.0 | 356 | 290.0 | No | 83.0 | 356 | 1.8265 | 0.745091 | 3.2330 | No | |
| 4 | SASDAZ3 | SASBDB | 80.0 | 280 | 0.0 | No | 80.0 | 280 | 1.812771 | 0.740641 | 2.8414 | No | |
| 5 | SASDA65 | SASBDB | 52.0 | 0.474 | 167.0 | No | 52.0 | 0.474 | 1.6027 | 0.741047 | 2.7473 | No | |
| 5 | SASDA74 | SASBDB | 24.0 | 207 | 85.0 | No | 24.0 | 207 | 2.962136 | 0.774485 | 1.8404 | **Yes** | |
| 5 | SASDAP4 | SASBDB | 32.0 | 2850 | 110.0 | No | 32.0 | 2850 | 2.0000 | 0.75656 | 2.3352 | **Yes** | |
| 6 | SASDA37 | SASBDB | 47.2 | 4140.4 | 190.0 | No | 47.2 | 4140.4 | 6.3091 | 0.772973 | 8.8173 | **Yes** | Elongated |
| 6 | SASDA47 | SASBDB | 77.0 | 7363.77 | 305.0 | No | 77.0 | 7363.77 | 10.5351 | 0.815768 | 11.6919 | **Yes** | Elongated |
| 6 | SASDA57 | SASBDB | 97.0 | 8587.8 | 385.0 | No | 97.0 | 8587.8 | 11.3802 | 0.800121 | 18.0543 | **Yes** | Elongated |
| 6 | SASDA67 | SASBDB | 120.0 | 9924.5 | 480.0 | No | 120.0 | 9924.5 | 10.4013 | 0.815834 | 21.5990 | **Yes** | Elongated |
| 6 | SASDA77 | SASBDB | 141.0 | 10488.6 | 570.0 | No | 141.0 | 10488.6 | 30.2271 | 0.783354 | 30.3913 | **Yes** | Elongated |

| | | | | | | | | | | | | | |
|---|---|---|---|---|---|---|---|---|---|---|---|---|---|
| 6 | SASDA87 | SASBDB | 159.0 | 14578 | 630.0 | No | 159.0 | 14578 | 35.8846 | 0.789852 | 29.1923 | **Yes** | Elongated |
| 6 | SASDAA4 | SASBDB | 39.0 | 160 | 145.0 | No | 39.0 | 160 | 1.773824 | 0.750228 | 5.7315 | No | |
| 6 | SASDA56 | SASBDB | 40.0 | 28 | 145.0 | No | 40.0 | 28 | 1.9554 | 0.763189 | 6.5088 | **Yes** | Elongated |
| 6 | SASDA66 | SASBDB | 42.0 | 37.5 | 160.0 | No | 42.0 | 37.5 | 2.2238 | 0.765393 | 6.6023 | **Yes** | Elongated |
| 7 | SASDA54 | SASBDB | 28.0 | 225.0 | 100.0 | No | 28.0 | 225.0 | 2.0478 | 0.803131 | 1.9935 | **Yes** | |
| 7 | SASDAU5 | SASBDB | 43.0 | 2365600.0 | 160.0 | Yes (2.01, 0.79) | 38.2 | 2230000 | 1.585305 | 0.743525 | 2.8308 | No | |
| 7 | SASDA88 | SASBDB | 27.0 | 0.0 | 100.0 | No | 27.0 | 0.0 | 2.897361 | 0.768371 | 2.1630 | **Yes** | |
| 7 | SASDA86 | SASBDB | 40.0 | 1270.0 | 140.0 | No | 40.0 | 1270.0 | 1.774991 | 0.772738 | 2.4964 | **Yes** | |
| 7 | 1U2FKX | BIOISIS | 32.1 | 349.0 | 115.0 | No | 32.1 | 349.0 | 1.7881 | 0.746182 | 3.0494 | No | |
| 7 | MnmGEP | BIOISIS | 67.4 | 0.2 | 236.0 | No | 67.4 | 0.2 | 1.6654 | 0.744035 | 2.7329 | No | |
| 7 | SASDAK5 | SASBDB | 40.0 | 10070.0 | 145.0 | Yes (2.24, 0.78) | 35.7 | 9530.0 | 1.783373 | 0.735475 | 3.8135 | No | |
| 7 | SASDAE4 | SASBDB | 55.0 | 10500.0 | 200.0 | No | 55.0 | 10500.0 | 2.2760 | 0.747438 | 5.4312 | **Yes** | |
| 7 | SASDA26 | SASBDB | 32.0 | 1284.0 | 110.0 | No | 32.0 | 1284.0 | 1.910251 | 0.757498 | 4.0485 | No | |
| 7 | SASDAB4 | SASBDB | 36.0 | 169.0 | 125.0 | No | 36.0 | 169.0 | 2.0663 | 0.75174 | 4.5736 | **Yes** | |
| 7 | SASDAS6 | SASBDB | 44.0 | 23.7 | 145.0 | No | 44.0 | 23.7 | 2.6066 | 0.779872 | 5.9151 | **Yes** | |
| 7 | SASDA76 | SASBDB | 51.0 | 1370.0 | 170.0 | No | 51.0 | 1370.0 | 2.5866 | 0.804183 | 2.7228 | **Yes** | |
